# The Potential of the Probiotic Isolate *Lactobacillus plantarum* SS18‐50 to Prevent Colitis in Mice

**DOI:** 10.1002/fsn3.4657

**Published:** 2024-12-09

**Authors:** Dan Wang, Tiehua Zhang, Luxin Qiu, Changhui Zhao

**Affiliations:** ^1^ Changchun Polytechnic Changchun China; ^2^ College of Food Science and Engineering Jilin University Changchun China

**Keywords:** isolates from space traveling, *Lactobacillus plantarum*, ulcerative colitis

## Abstract

The objective of this study was to investigate the effect of the *Lactobacillus plantarum* ( *L. plantarum* ) SS18‐50 (an isolate with favorable probiotic properties following space traveling) on dextran sulfate sodium (DSS)‐induced colitis in mice. Male ICR mice were randomly assigned to one of six groups: a control group, a model group, and four intervention groups comprising the isolate (SS18‐50‐L and SS18‐50‐H) and the wild type (GS18‐L and GS18‐H) strains. The model group and the intervention groups were administered a 3.5% DSS (w/v) solution to induce acute enteritis. The four intervention groups were administered the corresponding bacterial suspensions, SS18‐50‐L (1.0 × 10^7^ CFU/mL), SS18‐50‐H (1.0 × 10^9^ CFU/mL), GS18‐L (1.0 × 10^7^ CFU/mL), and GS18‐H (1.0 × 10^9^ CFU/mL). The results demonstrated that the disease activity index (DAI) score of the SS18‐50‐H was markedly lower than that of the CON. Subsequently, the colon tissue of mice was analyzed to determine the levels of myeloperoxidase (MPO), superoxide dismutase (SOD), glutathione (GSH), and malondialdehyde (MDA). The results demonstrated that all strains within the intervention groups exhibited good performance to prevent colitis. Particularly, the SS18‐50‐H strain exhibited a pronounced stimulative effect on GSH, an increase in SOD activity, and a decrease in MPO activity and MDA content. The SS18‐50‐H treatment resulted in a notable elevation in serum somatostatin (SS) levels and a concomitant reduction in endothelin (ET) and substance P (SP) levels, which approached normal ranges. The results of the RT‐qPCR analysis demonstrated that the mRNA expression levels of tumor necrosis factor (TNF‐α), cyclooxygenase (COX‐2), interleukin (IL‐10), and interleukin (IL‐6) in the SS18‐50‐H were significantly reduced to levels comparable to those observed in the CON. In conclusion, *L. plantarum* SS18‐50 has been demonstrated to inhibit the development of colitis in a dose‐dependent manner, thereby establishing it as a high‐quality lactic acid bacterium with a colitis inhibitory effect.

## Graphical abstract

The pioneered use of space radiation resulted in a novel probiotic strain *L. plantarum* SS18‐50 with superior probiotic properties. SS18‐50 effectively inhibited colitis in mice, reducing gut damage, decreasing inflammatory cytokine levels, and alleviating oxidative stress in a dose‐dependent manner. Therefore, *L. plantarum* SS18‐50 is a promising lactic acid bacterium with anti‐colitis properties that may be used as a functional food ingredient.

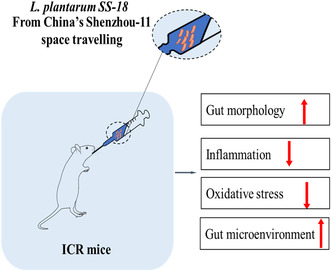

## 1 Introduction

Probiotics are defined as “live microorganisms which when administered in adequate amounts confer a health benefit on the host” (Hill et al. 2014). Probiotics can be utilized in the production of food and pharmaceuticals with probiotic functionality. The global probiotics market produced values of approximately 60 billion dollars in 2021 and is projected to reach close to 100 billion by 2026 (Mulay et al. 2022).

A number of studies have demonstrated the health benefits of probiotics, which include the inhibition of harmful bacteria in the intestine, contribution to the digestion and absorption of nutrients, regulation of the immune function of the body, promotion of the toxic substance decomposition in the intestine, resistance to allergies, prevention of hypertension, hyperlipidemia, hyperglycemia, and prevention of colon cancer (Kim et al. 2019; Sun et al. 2023). Recently, the scientific community has witnessed a surge in the discovery of numerous novel probiotic strains that exhibit remarkable potential in mitigating the symptoms of colitis and associated inflammatory phenotypes. This exciting development has been documented in several studies, which highlight the ability of these newly identified probiotics to alleviate inflammatory bowel disease (IBD) and potentially contribute to improved gastrointestinal health (Cordeiro et al. 2021; Song et al. 2023). Consequently, researchers in the food and pharmaceutical fields are highly interested in probiotics with excellent probiotic properties.

Probiotics are derived primarily from fermented foods, as well as from humans, animals, plants, and the ocean. The isolation and screening of probiotics from these living environments represents the most direct and effective method of probiotics mining. An alternative approach is to obtain probiotics with excellent probiotic properties through mutation breeding. Conventional mutagenic breeding employs physical or chemical techniques to induce mutations in bacteria, typically utilizing ultraviolet radiation and chemical agents. The success of space mutagenesis in crop breeding indicates that this method can be used as a new means of microbial breeding. The primary factors responsible for genetic polymorphisms and the production of genetically modified organisms are DNA damage and chromosomal aberrations caused by cosmic radiation. These changes can be exploited to develop the next generation of organisms capable of surviving under disparate environmental conditions.

In this study, the wild‐type *L. plantarum* was sent into outer space by the Shenzhou‐11 spacecraft at an altitude of 393 km for 31 days and 18.5 h. Following its return to Earth, the isolates from space with excellent probiotic properties including milk fermentation performance, low pH resistance, bile salt tolerance, hydrophobicity, and antibacterial activity were screened through in vitro and in vivo experiments (Wang et al. 2022; Wang et al. 2020).

The intestinal tract is the body's largest site for nutrient digestion and absorption (Wang et al. 2024). However, the intestinal tract is easily affected by the environment, diet, and medication and can cause inflammation. The prevalence and incidence of IBD have been increasing, affecting hundreds of people worldwide (Kai et al. 2024). Ulcerative colitis (UC) is a common research object in IBD. It is a chronic intestinal disease that is prone to relapse. UC is a lifelong inflammatory disease that affects the rectum and colon to varying degrees, which can be caused by bacteria, fungi, viruses, parasites, protozoa, as well as allergic reactions and physicochemical factors. UC poses problematic issues to society, including impacts on individual health, economic burdens, social and psychological impacts, and increased healthcare burdens (Ng et al. 2017). In 2023, the prevalence of UC is estimated to be 5 million cases worldwide, and the incidence is still rapidly increasing worldwide (Le Berre, Honap, and Peyrin‐Biroulet 2023). Despite the expansion of modern therapeutic options, a significant proportion of patients (10%–20%) still require proctocolectomy due to the persistence of medically refractory disease (Le Berre, Honap, and Peyrin‐Biroulet 2023).

Gastrointestinal regulatory peptides related to enteritis include substance P (SP), endothelin (ET‐1), somatostatin (SS), and vasoactive intestinal peptide (VIP). Studies (Patel et al. 2020) have shown that SP has a proinflammatory effect and its level is positively correlated with the degree of disease activity in IBD. ET‐1, a vasoconstrictor and chemoattractant, exacerbates tissue damage associated with IBD and is significantly higher in IBD patients than in controls (Angerio et al. 2005). On the contrary, SS and VIP have anti‐inflammatory effects and their levels are lower in the serum of IBD patients than in controls (El‐Salhy et al. 2017; Sun et al. 2019).

The pathological progression of UC is related to the abnormal generation of reactive oxygen species (ROS) (Zhu and Li 2012). When an excess of ROS is produced in cells, aberrant alterations in chemical composition and chemical reactions at all levels may ensue, ultimately culminating in cell death. Superoxide dismutase (SOD) and glutathione (GSH) are two intracellular antioxidants that are capable of removing excess ROS and maintaining intracellular ROS balance (Chen et al. 2023). Myeloperoxidase (MPO) is associated with ROS production (Kim et al. 2012) and malondialdehyde (MDA) is the end product of oxidative reactions, both of which can be used as markers of oxidative stress (Cui et al. 2018).

UC is associated with the expression of many inflammation‐related genes. TNF‐α (tumor necrosis factor‐α) is a proinflammatory cytokine. COX‐2 (cyclooxygenase‐2) is a key enzyme that catalyzes the conversion of arachidonic acid to prostaglandins, which plays an important role in colonic mucosal defense (Zizzo et al. 2020). The expression levels of TNF‐α and COX‐2 were positively correlated with the severity of UC (Youn et al. 2024). In the colitis model, IL‐6 (interleukin‐6) expression was increased, while its antibody showed an inhibitory effect on colitis (Sommer et al. 2014). IL‐10 (interleukin‐10) is an anti‐inflammatory cytokine secreted by T cells and B cells, which can inhibit the expression of cytokines, and its expression was also increased in UC patients (Dayagi et al. 2021).

There is an imbalance of commensal microorganisms in UC patients and animal models (Alam et al. 2020). Therefore, when the body undergoes enteritis, the overgrowth and multiplication of harmful microorganisms invade the damaged epithelial barrier, which leads to the aggravation of the disease (Hansen 2015). Clinically, the regulation of gut microbiota is considered as a potential target for the prevention and treatment of UC. A growing body of literature has shown that probiotics are closely related to human health, especially intestinal health (Kim et al. 2019). Meta‐analysis has shown that probiotics are effective and safe for the prevention of recurrence of UC (Derwa et al. 2017). Probiotics have immunomodulatory effects, improve the function of intestinal barrier, and maintain the balance of the body and intestinal microorganisms (Rodriguez‐Nogales et al. 2017). A variety of *L. plantorum* has been demonstrated to alleviate the symptoms of UC through the maintenance of intestinal stability, the downregulation of proinflammatory cytokines in serum, and other mechanisms (Jin et al. 2020; Niu et al. 2023; Sun et al. 2020). The administration of these probiotics may result in the alleviation of symptoms associated with UC by promoting the growth of beneficial microbiota while simultaneously reducing the population of harmful bacteria that contribute to the progression of UC.

Our previous in vitro and in vivo experiments show that the space‐mutated *L. plantarum* SS18‐50 strain has better gastrointestinal tolerance and antibacterial activity than the ground‐based wild strain GS18. We hypothesize that these excellent probiotic properties confer the ability of *L. plantarum* SS18‐50 to modulate gut microbiota imbalance in UC. Therefore, the aim of the present research is to investigate the therapeutic effect of this strain of SS18‐50 on dextran sulfate sodium (DSS)‐induced colitis in mice.

## 2 Materials and Methods

### 2.1 Bacterial Strains and Animals

The strains used in this experiment were *L. plantarum* GS‐18 and *L. plantarum* SS18‐50, which were provided by Beijing Fulerton BioEngineering Co. Ltd. The wild‐type *L. plantarum* GS‐18 carried by the Shenzhou‐11 spacecraft (China National Space Administration) was sent into outer space at an altitude of 393 km and then traveled in space for 31 days and 18.5 h. Following its return to Earth, the isolates from space traveling with excellent probiotic properties including good milk fermentation performance, low‐pH resistance, high bile salt tolerance, hydrophobicity, and antibacterial activity were screened out through in vitro experiments to obtain *L. plantarum* SS18‐50 (Wang et al. 2022; Wang et al. 2020). Forty‐eight male ICR mice (SPF, weighing 20 ± 2 g) were purchased from Liaoning Changsheng Biotechnology Co. Ltd.

### 2.2 Preparation of Bacterial Bodies by Gavage in Mice

*L. plantarum* GS‐18 and *L. plantarum* SS18‐50 were cultured in a modified MRS medium at 37°C for a period of 18 h. The number of viable bacteria was adjusted to 1.0 × 10^9^ CFU/mL and subjected to centrifugation (6000 g, 10 min, 4°C). The resulting precipitate was washed twice with 0.85% sterile sodium chloride solution to remove residual medium and then suspended in 0.85% sterile sodium chloride solution until further use.

### 2.3 Experimental Design

**FIGURE 1:**
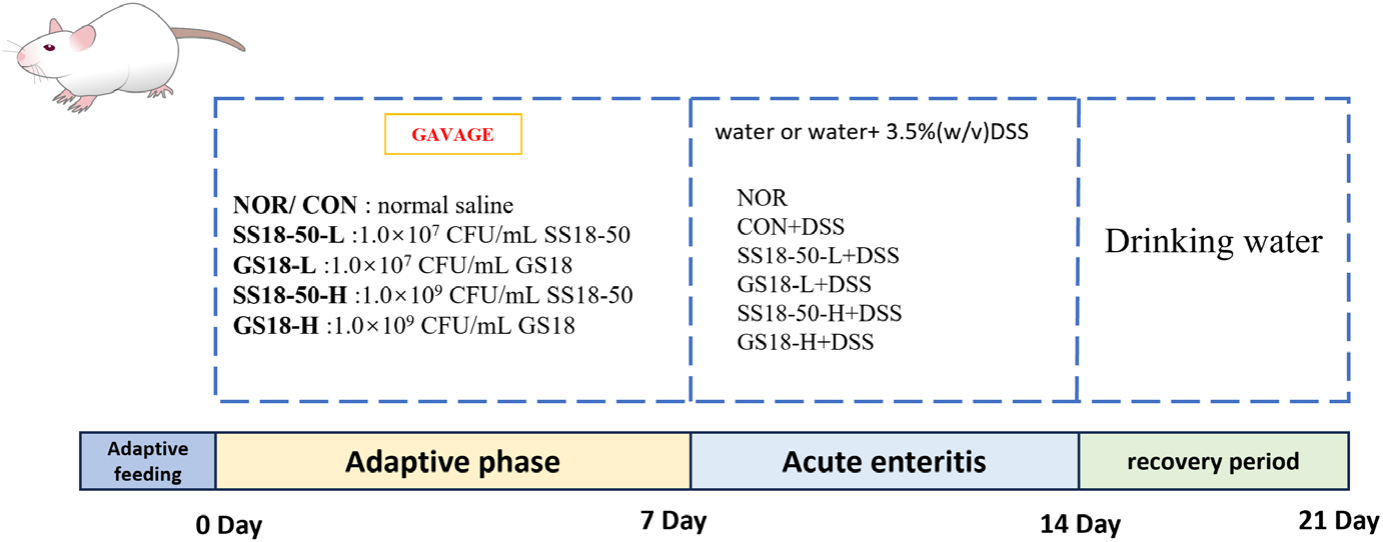
Experimental design diagram.

A total of 48 male ICR mice were provided with *ad libitum* access to food and water and maintained at a temperature of 23°C ± 1°C, humidity of 55% ± 5%, and 12/12 h light–dark cycles with alternating light and dark conditions. Following a one‐week period of acclimatization prior to the commencement of the experiment, the mice (eight mice in each group) were randomly assigned to one of six groups: the normal group (NOR), the DSS‐induced enteritis model group (CON), the low‐dose space *L. plantarum* SS18‐50 group (SS18‐50‐L, 1.0 × 10^7^ CFU/mL), the high‐dose space *L. plantarum* SS18‐50 group (SS18‐50‐H), the low‐dose wild type *L. plantarum* GS18‐50 group (GS18‐50‐L, 1.0 × 10^7^ CFU/mL), and the high‐dose wild type *L. plantarum* GS18‐50 group (GS18‐50‐H, 1.0 × 10^9^ CFU/mL). The experiment was conducted over a period of 21 days, divided into three phases: the initial adaptation period (days 1–7), the subsequent acute enteritis period (days 8–14), and the final recovery period from enteritis (days 15–21). The gavage dose was 10 mL per kilogram of body weight per animal per day. The NOR was administered sterile saline via gavage on a daily basis throughout the course of the experiment. During the initial adaptation period (days 1–7), the mice in the CON were administered sterile saline via gavage, while the mice in the four strain intervention groups (SS18‐50‐L, GS18‐L, SS18‐50‐H, and GS18‐H) received gavage with the corresponding concentrations of bacterial suspension. During the second phase of acute enteritis (8–14 days), the NOR was provided with regular drinking water. The drinking water of the control and strain intervention groups was replaced with 3.5% (w/v) DSS (purchased from Beijing Coolibo Biotechnology Co. Ltd.), and the strain intervention group was administered the corresponding bacterial suspension via gavage. In the third stage of enteritis recovery (15–21 days), all DSS solutions were returned to normal water, and the strain intervention group continued to administer the corresponding bacterial suspension via gavage. The detailed experimental design is shown in Figure 1. This animal experimental protocol (20161013) was reviewed and approved by the Animal Ethics Committee of Jilin University.

### 2.4 Determination of the Score of the Disease

In the experiment, the mice were weighed every day. The body weight was measured every day. And the stool shape as well as the anal bleeding were recorded accordingly. The disease activity index (Camuesco et al. 2004; Cooper et al. 1993) (DAI) was used to calculate the score of the disease, namely DAI = (body mass index + stool shape + bleeding). The specific score details are shown in Table 1.

**TABLE 1:** Scoring criteria for disease activity index.

Score	Weight loss percentage	Fecal consistency	Stool bleeding
0	0	Normal	Normal
1	1%–5%	—	—
2	5%–10%	Loose stool	Occult blood test
3	10%–20%	—	—
4	> 20%	Diarrhea	Revealed hemorrhage

### 2.5 Determination of MPO, SOD, GSH, and MDA in Colonic Tissues

The tissue sample (approximately 0.1 g) was weighed and then immersed in 1 mL of extraction solution to perform grinding in an ice bath. Subsequently, the homogenate was centrifuged at 8000 g at 4°C for 10 min. After centrifugation, the supernatant was used for determination. The levels of MPO, SOD, GSH, and MDA in colonic tissue of mice were quantified in accordance with the instructions provided with the commercial kits (Nanjing Jiechan Bioengineering Institute).

### 2.6 The Determination of Gastrointestinal Regulatory Peptide in the Serum

The tissue sample (0.1 ‐ 0.2 g) was rinsed on iced with saline to remove blood then dried with filter paper and placed in a homogenizing tube. After adding 9 times the volume of physiological saline based on the weight‐to‐volume ratio (g:mL), the tissue was thoroughly ground into homogenate under ice‐water bath conditions. The prepared 10% homogenate was centrifuged at 1000 g for 10 min. The supernatant was collected for measurement. The concentrations of gastrointestinal regulatory peptides, including SP, VIP, SS, and ET‐1, were quantified in mouse serum using commercial ELISA kits (Nanjing Jianxian Bioengineering Institute), in accordance with the kit instructions.

### 2.7 Real‐Time Quantitative PCR (RT‐qPCR)

The total RNA was initially extracted from the colon using the Trizol reagent (Thermo Fisher Scientific, USA). DSS inhibited the amplification of RT‐qPCR, so lithium chloride method was used to remove the residual DSS in RNA (Viennois et al. 2013). Purified RNA was reversed into cDNA using the All‐In‐One 5X RT MasterMix (G592, applied Biological Materials, Canada). qPCR amplification was then performed using BlasTaq 2X qPCR MasterMix (G891, purchased from Applied Biological Materials, Canada), and the primers (purchased from Sunon Bioengineering (Shanghai) Co. Ltd.) are shown in Table 2. Gene expression levels were calculated using the △△^C^_t_ method and normalized to the internal reference β‐actin.

**TABLE 2:** Primer sequences of mRNA for RT‐qPCR.

Item	Primer sequence(5′–3′)	Reference
β‐Actin	F: AGAGGGAAATCGTGCGTGAC R: CAATAGTGATGACCTGGCCGT	(Lee et al. 2009)
TNF‐α	F: CATCTTCTCAAAATTCGAGTGACAA	(Lee et al. 2009)
	R: TGGGAGTAGACAAGGTACAACCC	
COX‐2	F: GGTGGAGAGGTGTATCCCCC	(Lee et al. 2009)
	R: ACTTCCTGCCCCACAGCA	
IL‐6	F: GTTCTCTGGGAAATCGTGGA	(Dombrowicz et al. 2001)
	R: TGTACTCCAGGTAGCTATGG	
IL‐10	F: ATGCAGGACTTTAAGGGTTACTTG	(Dombrowicz et al. 2001)
	R: AGACACCTTGGTCTTGGAGCTTA	

### 2.8 Statistical Analysis

The experimental data are expressed as mean ± standard deviation. Statistical charts were generated with Origin 8.5 software. One‐way ANOVA was performed followed by Duncan's *post hoc* analysis using SPSS 19. A significant difference was considered when *p* < 0.05.

## 3 Results

### 3.1 The Macroscopic Evaluation of Mice

**FIGURE 2:**
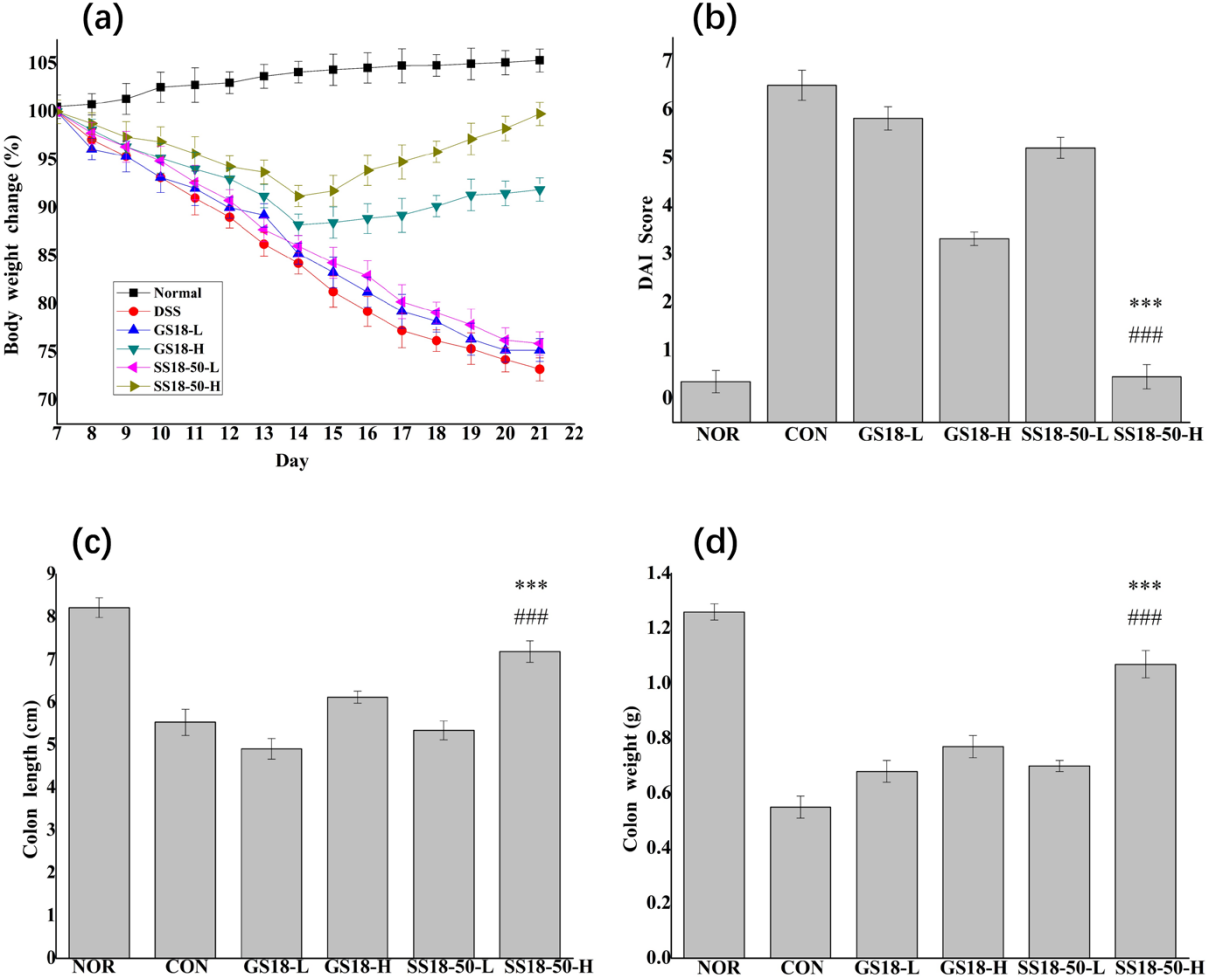
Effect of strain intervention on colitis mice. (a) The change of body weight; (b) DAI score during recovery of enteritis; (c) colon length; (d) colon weight; versus GS18‐H, ***p < 0.001; versus CON, ###*p* < 0.001.

The severity of colitis in mice is typically assessed through the measurement of weight loss and the calculation of a DAI score (Zhang et al. 2017). A successful UC model with a low DAI score can be constructed by using 3% (w/v) DSS as drinking water for 7 days (Gao et al. 2016; Wakuda et al. 2013). Indeed, following the administration of DSS on day 8, the body weight of mice in the CON and the intervention groups with varying doses of strains (GS18 and SS18‐50) exhibited a notable decline (Figure 2a). On the removal of DSS on day 14, the GS18‐H and SS18‐50‐H entered the recovery phase of colitis, exhibiting a rapid recovery in body weight. On the final day of the experiment (day 21), the body weight of the SS18‐50‐H was observed to be approaching that of the NOR. The DAI score of the SS18‐50‐H was significantly lower than that of the CON (*p* < 0.001), and was comparable to that of the NOR (Figure 2b). The colon length in the CON was shorter and the weight decreased, indicating that the colitis model had been successfully established (Figure 2c,d) Significant differences in colon length and weight were observed between the SS18‐50‐H and GS18‐H (*p* < 0.05). The SS18‐50‐H and NOR exhibited similar characteristics. These findings suggest that SS18‐50‐H may possess the capacity to avert DSS‐induced colitis in mice.

### 3.2 Determination of MPO, MDA, GSH, and SOD in the Colon

**FIGURE 3:**
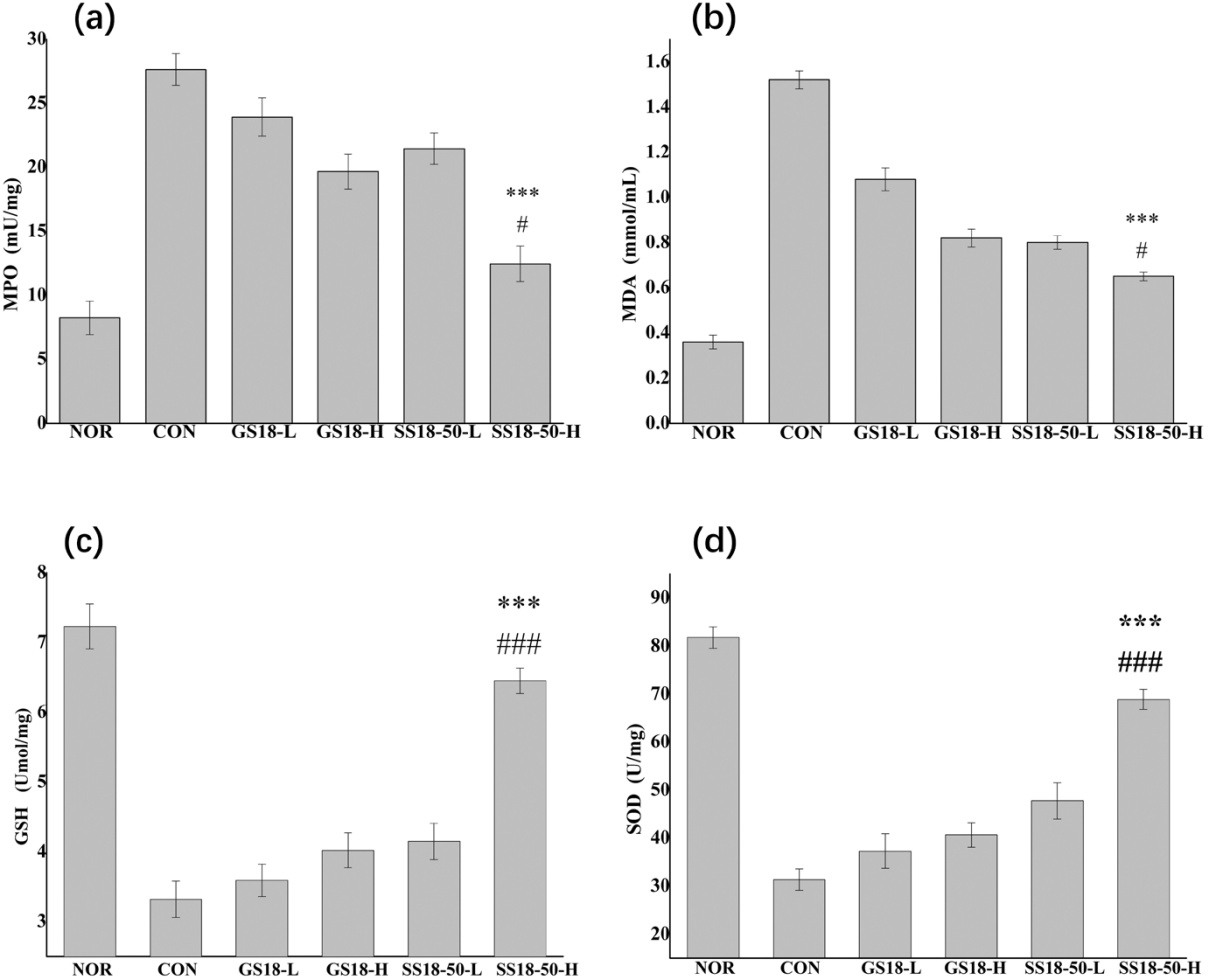
MPO, GSH, MDA, and SOD colon tissue contents in mice. (a) MPO; (b) MDA; (c) GSH; (d) SOD. Versus CON, ***p < 0.001, versus GS18‐H, # *p* < 0.05, ### *p* < 0.001.

The NOR exhibited the highest GSH content, the strongest SOD activity, the lowest MPO activity, and the weakest MDA content. This allows for the maintenance of normal oxidation levels within the body and prevents the damage caused by free radicals (Figure 3). A significant decrease in GSH content and SOD activity was observed in the CON when compared to the NOR (*p* < 0.05). Conversely, a significant increase in MPO activity and MDA content was noted in the CON when compared to the NOR (*p* < 0.05). These findings indicate that the enteritis model was successfully established (Figure 3a–d). The administration of different doses of the strain intervention groups (GS18 and SS18‐50) resulted in an increase in GSH content, an increase in SOD activity, a decrease in MPO activity, and a decrease in MDA content when compared to the CON. The SS18‐50‐H exhibited superior performance, with a more pronounced difference (*p* < 0.001), approaching the levels observed in the NOR.

### 3.3 Measurement of Gastrointestinal Regulatory Peptides in Serum

**FIGURE 4:**
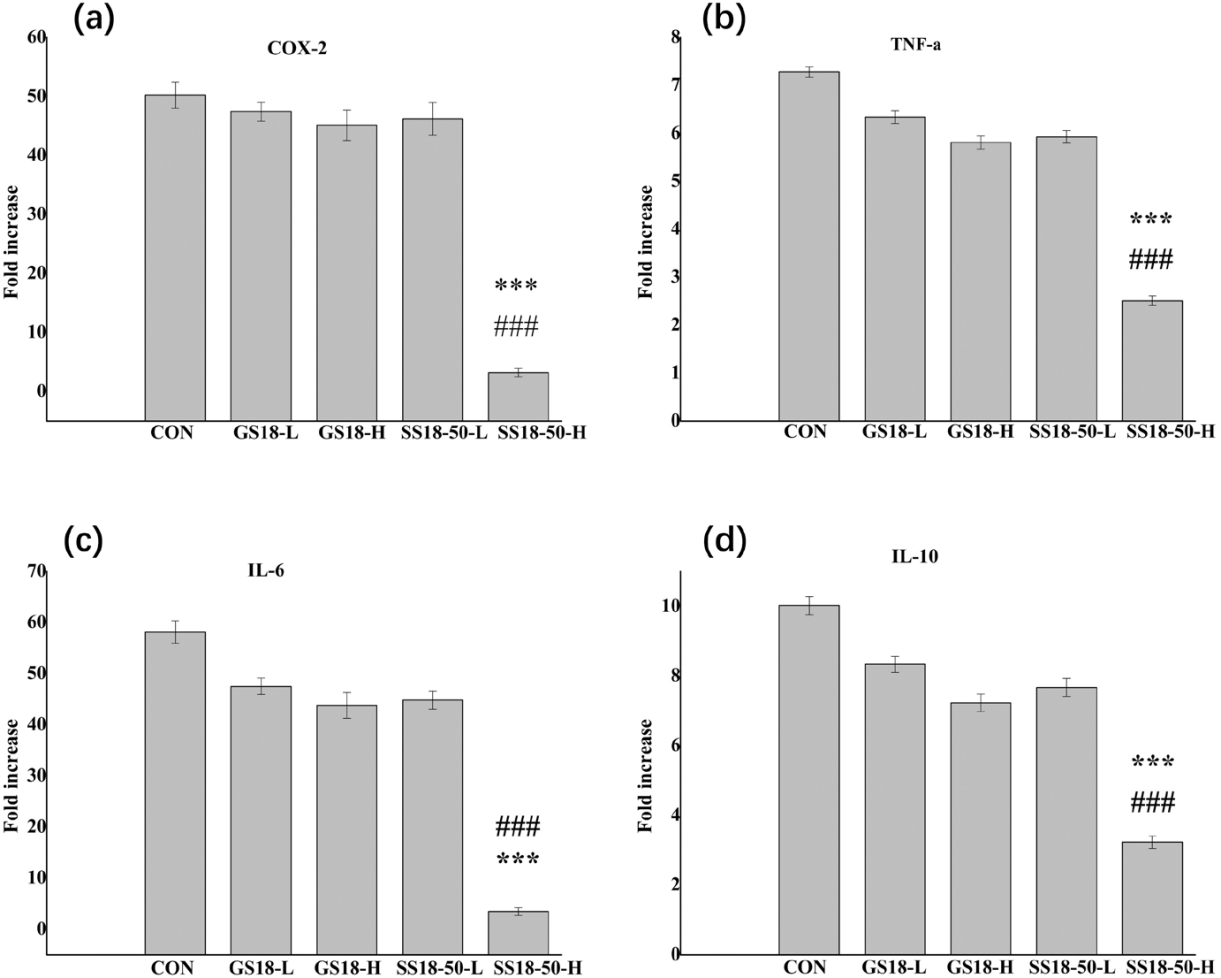
ET, SS, SP, and VIP serum levels in mice. (a)ET‐1; (b) SS; (c) SP; (d) VIP; versus GS18‐H, ***p < 0.001, versus CON, ###*p* < 0.001.

The levels of SS and VIP in the serum of the NOR were found to be higher than those observed in the other groups, while the levels of ET and SP were observed to be lower than those observed in the other groups (Figure 4). The levels of ET and SP in the CON were the highest, while the levels of SS and VIP were the lowest. This result indicated that the mouse enteritis model was successfully established. In comparison to the CON, SS18‐50‐H treatment resulted in a notable elevation in SS and VIP levels (*p* < 0.05), accompanied by a pronounced reduction in ET and SP levels (*p* < 0.05). These outcomes were observed to be approaching those of the NOR. Consequently, SS18‐50‐H demonstrated a more pronounced advantage in terms of gastrointestinal regulatory peptides when compared to GS18‐H.

### 3.4 The Expression of Genes Associated With Inflammation

**FIGURE 5:**
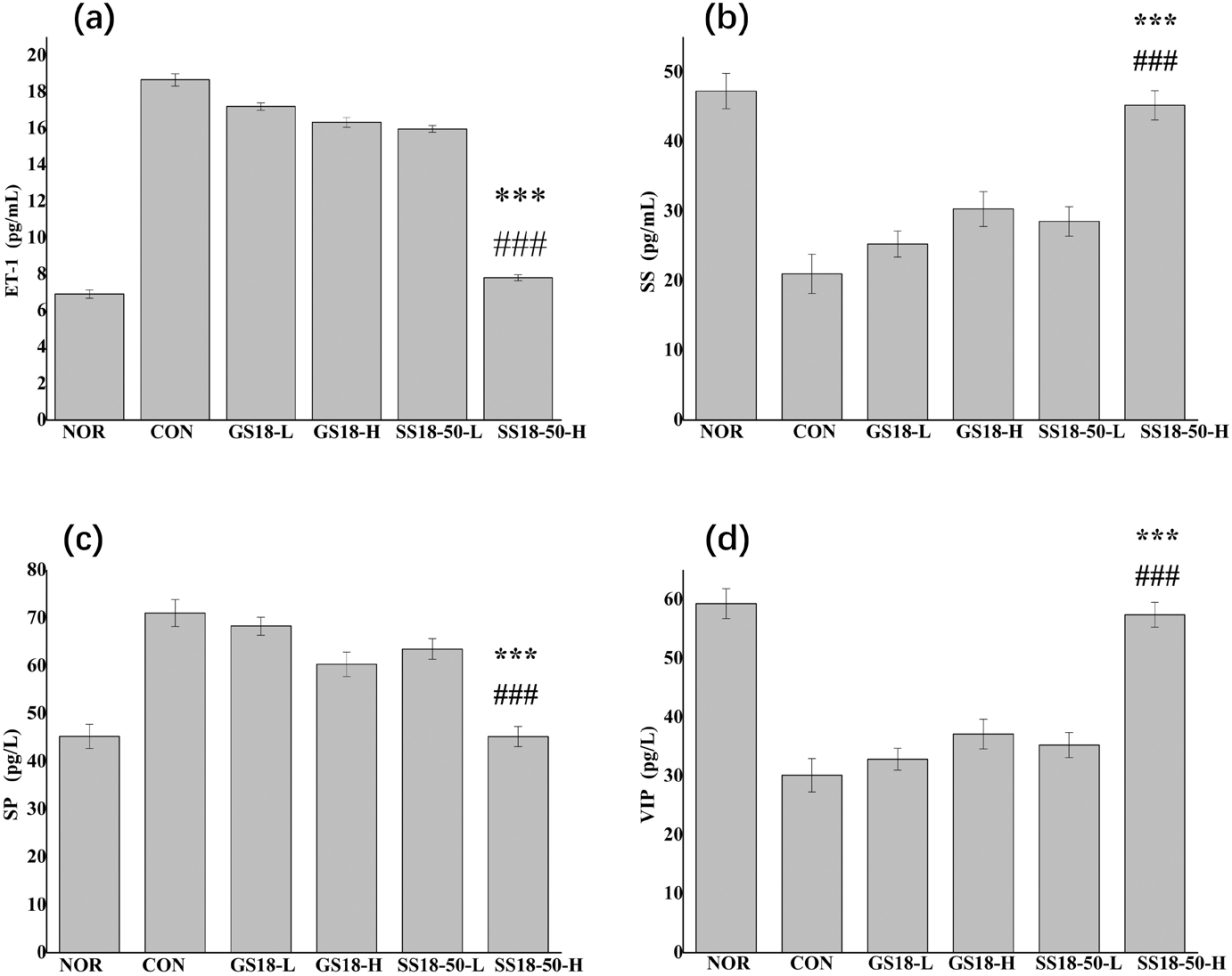
Effects of *L. plantarum* SS18‐50 and GS18 on mRNA expression in the colons. (a) COX‐2; (b) TNF‐α; (c) IL‐6; (d) IL‐10; versus GS18‐H, ***p < 0.001, versus CON, ###*p* < 0.001.

The expression of colitis‐related genes, including COX‐2, TNF‐α, IL‐6, and IL‐10, was detected in the colon using RT‐qPCR. Following the administration of DSS, the CON, GS18‐L, GS18‐H, and SS18‐50‐L demonstrated a notable elevation in the relative mRNA expression of TNF‐α, COX‐2, IL‐10, and IL‐6, respectively (Figure 5). In comparison to the NOR, the relative mRNA expression of TNF‐α, COX‐2, IL‐10, and IL‐6 was observed to be increased by 7‐, 49‐, 10‐, and 51‐fold, respectively, in the CON, GS18‐L, GS18‐H, and SS18‐50‐L (*p* < 0.01). However, the mRNA expressions of TNF‐α, COX‐2, IL‐10, and IL‐6 in the SS18‐50‐H were observed to return to a level comparable to that of the NOR.

## 4 Discussion

A substantial body of evidence indicates that probiotics are frequently employed in the development of functional foods with the objective of enhancing intestinal health. The potential of probiotics as a means of preventing and treating UC has attracted increasing attention from scholars. The objective of the current study was to evaluate the effect of *L. plantarum* SS18 with excellent probiotic properties on DSS‐induced colitis in mice. The DSS‐induced colitis model closely resembles the clinical manifestations of human UC (Valatas, Vakas, and Kolios 2013).

UC is a typical IBD that has the potential to significantly impact an individual's quality of life and may also give rise to a range of other health complications. It is unfortunate that the prevalence of UC is increasing worldwide, representing a significant public health concern that requires the attention of both researchers and healthcare systems. Prophylactic interventions and oral supplementation of probiotics in animals have been demonstrated to be more effective in the recovery period of colitis (Burns et al. 2017; Hayes et al. 2014). The impact of probiotics on UC was dependent on the specific strain (Burns et al. 2017). For example, *Lactobacillus reuteri* F‐9‐35, which was also isolated from space traveling showed a good preventative effect on DSS‐induced colitis by inhibiting pro‐inflammatory markers and demodulating the gut microbiota in mice (Sun et al. 2018). Similarly, in our study, *L. plantarum* SS18‐50 demonstrated superior efficacy in the prevention of colitis in mice compared to the wild‐type ground strain GS18. The administration of high concentrations of strains has been demonstrated to facilitate the recuperation of UC. In the event of inflammation in mouse colon lesions, neutrophils in the affected tissues aggregate and begin to decrease, while a considerable number of neutrophils enter the tissues, resulting in a rapid increase in MPO activity (Mustafa et al. 2006; Zheng et al. 2016). A substantial quantity of free radicals accumulates, resulting in irreversible damage to colonic tissues (Osman et al. 2008). However, the incidence of colitis will result in a notable reduction in GSH content and SOD activity, while MDA levels will increase considerably (Hu et al. 2021; Zhang et al. 2023). It is, therefore, essential to regulate the aggregation of free radicals in tissues and enhance the activity of enzymes that inhibit oxidation, in order to play a role in inhibiting colitis. The findings of our study corroborate the notion that UC is associated with a decline in SOD activity and GSH content, accompanied by an increase in MPO activity and MDA levels (Figure 3). *L. plantarum* SS18‐H has been demonstrated to exert a pronounced inhibitory effect (*p* < 0.05) on the oxidative stress response in the colonic mucosa of patients with UC, effectively alleviating the clinical manifestations of colitis.

Our study also investigated the differences between *L. plantarum* GS18 and *L. plantarum* SS18‐50 in regulating gastrointestinal regulatory peptides in the serum of mice with enteritis. The gastrointestinal regulatory peptides that are primarily associated with enteritis include SP, ET‐1, SS, and VIP. Of these, SP is an excitatory transmitter, whereas ET‐1, SS, and VIP are inhibitory transmitters. Excitatory transmitters primarily facilitate gastric contraction, accelerate intestinal transit time, stimulate the secretion of gastric acid and pepsin, promote gastrointestinal smooth muscle contraction, and relaxation of the pyloric sphincter. Conversely, inhibitory transmitters elicit the opposite effects. The findings of the gastrointestinal regulatory peptide study presented here are in alignment with previous reports (Kanmani et al. 2011; Sim et al. 2015). The majority of the intervention groups exhibited a pattern of excitatory transmitter secretion that was both higher than that observed in the NOR and lower than that observed in the NOR. In contrast, the inhibitory transmitter secretion was lower than that observed in the NOR (Figure 4). These findings suggest that *L. plantarum* SS18‐50‐H significantly inhibited the occurrence of colitis in comparison to the wild‐type GS18‐H.

UC is characterized by the overexpression of inflammation‐related genes. TNF‐α is a proinflammatory cytokine that can induce apoptosis of epithelial cells, destroy epithelial cell barrier, and prolong inflammatory response (Wan et al. 2020). In the colitis model, there is an increase in IL‐6 (interleukin‐6) expression, and the antibody to this protein has been demonstrated to exert an inhibitory effect on colitis (Sommer et al. 2014). COX‐2 is a pivotal enzyme that facilitates the conversion of arachidonic acid to prostaglandins, which serve a crucial function in the defense of colonic mucosa. The expression level of COX‐2 mRNA has been demonstrated to be positively correlated with the severity of UC (Youn et al. 2024). IL‐10 is an anti‐inflammatory cytokine secreted by T cells and B cells, which plays a role in maintaining intestinal stability (Dayagi et al. 2021). The present study demonstrated that SS18‐50‐H treatment resulted in a reduction in mRNA expression of TNF‐α, COX‐2, IL‐10, and IL‐6 to levels within the normal range in colitis mice (Figure 5).

In summary, compared with GS18‐H, *L. plantarum* SS18‐50‐H exhibited a superior efficacy in treating colitis, as evidenced by the changes in body weight, colon length, and DAI score. In comparison to GS18‐H, *L. plantarum* SS18‐H demonstrated a notable capacity to impede colonic injury associated with UC and to mitigate colitis through the modulation of antioxidant defense indices, gastrointestinal regulatory peptides in serum, and inflammatory cytokines. It is noteworthy that the low‐dose group did not demonstrate a significant efficacy in alleviating the symptoms of UC. It can be stated that only the appropriate quantity of this probiotic is effective, which is in accordance with the definition of probiotics. A low dose is likely insufficient to alter the gut dysbiosis, wherein harmful bacteria predominate, a common occurrence in UC models. The 16S rDNA of L. plantorum was subjected to genotyping, which revealed the absence of sequence alterations (D. Wang et al. 2020). It is hypothesized that the therapeutic effect of this strain on UC is likely due to its probiotic properties, which include low pH resistance, bile salt tolerance, hydrophobicity, and antibacterial activity. These properties enable the beneficial strain to remain in the gut for an extended period, thereby maintaining optimal microbiota balance.

This study makes significant contributions to probiotics and gut health research through several key innovations. The pioneered use of cosmic radiation resulted in a novel probiotic strain *L. plantarum* SS18‐50 with superior probiotic properties. SS18‐50 effectively inhibited colitis in mice, reducing disease activity, tissue damage markers, and inflammatory cytokine levels in a dose‐dependent manner. Beyond efficacy, the study also evaluated the mechanisms underlying the probiotic effects. Therefore, *L. plantarum* SS18‐50 is a promising lactic acid bacterium with anti‐colitis properties that warrants further development and utilization.

## Author Contributions

**Dan Wang:** conceptualization (equal), investigation (equal), writing – original draft (equal). **Tiehua Zhang:** conceptualization (equal), supervision (equal). **Luxin Qiu:** writing – original draft (equal). **Changhui Zhao:** writing – review and editing (equal).

## Conflicts of Interest

The authors declare no conflicts of interest.

## Data Availability

The data that support the findings of this study are available on request from the first author.

## References

[fsn34657-bib-0001] Alam, M. T. , G. C. A. Amos , A. R. J. Murphy , S. Murch , E. M. H. Wellington , and R. P. Arasaradnam . 2020. “Microbial Imbalance in Inflammatory Bowel Disease Patients at Different Taxonomic Levels.” Gut Pathogens 12: 1–8. 10.1186/s13099-019-0341-6. 31911822 PMC6942256

[fsn34657-bib-0002] Angerio, A. D. , D. Bufalino , M. Bresnick , C. Bell , and S. Brill . 2005. “Inflammatory Bowel Disease and Endothelin‐1: A Review.” Critical Care Nursing Quarterly 28, no. 2: 208–213. 10.1097/00002727-200504000-00013. 15875451

[fsn34657-bib-0003] Burns, P. , J. Alard , J. Hrdy , et al. 2017. “Spray‐Drying Process Preserves the Protective Capacity of a Breast Milk‐Derived *Bifidobacterium lactis* Strain on Acute and Chronic Colitis in Mice.” Scientific Reports 7: 43211. 10.1038/srep43211. 28233848 PMC5324110

[fsn34657-bib-0004] Camuesco, D. , M. Comalada , M. E. Rodriguez‐Cabezas , et al. 2004. “The Intestinal Anti‐Inflammatory Effect of Quercitrin Is Associated With an Inhibition in iNOS Expression.” British Journal of Pharmacology 143, no. 7: 908–918. 10.1038/sj.bjp.0705941. 15533892 PMC1575937

[fsn34657-bib-0005] Chen, Y. , M. J. Shui , Q. Yuan , et al. 2023. “Wielding the Double‐Edged Sword: Redox Drug Delivery Systems for Inflammatory Bowel Disease.” Journal of Controlled Release 358: 510–540. 10.1016/j.jconrel.2023.05.007. 37169178

[fsn34657-bib-0006] Cooper, H. S. , S. N. Murthy , R. S. Shah , and D. J. Sedergran . 1993. “Clinicopathologic Study of Dextran Sulfate Sodium Experimental Murine Colitis.” Laboratory Investigation 69, no. 2: 238–249. 8350599

[fsn34657-bib-0007] Cordeiro, B. F. , J. L. Alves , G. A. Belo , et al. 2021. “Therapeutic Effects of Probiotic Minas Frescal Cheese on the Attenuation of Ulcerative Colitis in a Murine Model.” Frontiers in Microbiology 12: 623920. 10.3389/fmicb.2021.623920. 33737918 PMC7960676

[fsn34657-bib-0008] Cui, X. , J. Gong , H. Han , et al. 2018. “Relationship Between Free and Total Malondialdehyde, a Well‐Established Marker of Oxidative Stress, in Various Types of Human Biospecimens.” Journal of Thoracic Disease 10, no. 5: 3088–3197. 10.21037/jtd.2018.05.92. 29997978 PMC6006110

[fsn34657-bib-0009] Dayagi, T. W. , L. Werner , M. Pinsker , et al. 2021. “Mucosal IL‐10 and IL‐10 Receptor Expression Patterns in Paediatric Patients With Ulcerative Colitis.” International Journal of Experimental Pathology 102, no. 1: 4–10. 10.1111/iep.12382. 33405352 PMC7839950

[fsn34657-bib-0010] Derwa, Y. , D. J. Gracie , P. J. Hamlin , and A. C. Ford . 2017. “Systematic Review With Meta‐Analysis: The Efficacy of Probiotics in Inflammatory Bowel Disease.” Alimentary Pharmacology & Therapeutics 46, no. 4: 389–400. 10.1111/apt.14203. 28653751

[fsn34657-bib-0011] Dombrowicz, D. , S. Nutten , P. Desreumaux , et al. 2001. “Role of the High Affinity Immunoglobulin E Receptor in Bacterial Translocation and Intestinal Inflammation.” Journal of Experimental Medicine 193, no. 1: 25–34. 10.1084/jem.193.1.25. 11136818 PMC2195885

[fsn34657-bib-0012] El‐Salhy, M. , T. Solomon , T. Hausken , O. H. Gilja , and J. G. Hatlebakk . 2017. “Gastrointestinal Neuroendocrine Peptides/Amines in Inflammatory Bowel Disease.” World Journal of Gastroenterology 23, no. 28: 5068–5085. 10.3748/wjg.v23.i28.5068. 28811704 PMC5537176

[fsn34657-bib-0013] Gao, W. , Y. Guo , C. Wang , et al. 2016. “Indirubin Ameliorates Dextran Sulfate Sodium‐Induced Ulcerative Colitis in Mice Through the Inhibition of Inflammation and the Induction of Foxp3‐Expressing Regulatory T Cells.” Acta Histochemica 118, no. 6: 606–614. 10.1016/j.acthis.2016.06.004. 27396532

[fsn34657-bib-0014] Hansen, J. J. 2015. “Immune Responses to Intestinal Microbes in Inflammatory Bowel Diseases.” Current Allergy and Asthma Reports 15, no. 10: 61. 10.1007/s11882-015-0562-9. 26306907

[fsn34657-bib-0015] Hayes, C. L. , J. M. M. Natividad , J. Jury , R. Martin , P. Langella , and E. F. Verdu . 2014. “Efficacy of *Bifidobacterium breve* NCC2950 Against DSS‐Induced Colitis Is Dependent on Bacterial Preparation and Timing of Administration.” Beneficial Microbes 5, no. 1: 79–88. 10.3920/bm2013.0039. 24533977

[fsn34657-bib-0016] Hill, C. , F. Guarner , G. Reid , et al. 2014. “Expert Consensus Document. The International Scientific Association for Probiotics and Prebiotics Consensus Statement on the Scope and Appropriate Use of the Term Probiotic.” Nature Reviews. Gastroenterology & Hepatology 11, no. 8: 506–514. 10.1038/nrgastro.2014.66. 24912386

[fsn34657-bib-0017] Hu, T. T. , Y. Fan , X. Y. Long , et al. 2021. “Protective Effect of *Lactobacillus plantarum* YS3 on Dextran Sulfate Sodium‐Induced Colitis in C57BL/6J Mice.” Journal of Food Biochemistry 45, no. 2: e13632. 10.1111/jfbc.13632. 33527475

[fsn34657-bib-0018] Jin, J. , S. Wu , Y. Xie , H. Liu , X. Gao , and H. Zhang . 2020. “Live and Heat‐Killed Cells of *Lactobacillus plantarum* Zhang‐LL Ease Symptoms of Chronic Ulcerative Colitis Induced by Dextran Sulfate Sodium in Rats.” Journal of Functional Foods 71: 103994. 10.1016/j.jff.2020.103994.

[fsn34657-bib-0019] Kai, N. , C. Qingsong , M. Kejia , et al. 2024. “An Inflammatory Bowel Diseases Integrated Resources Portal (IBDIRP).” Database 2024: baad097. 10.1093/database/baad097. 38227799 PMC10791110

[fsn34657-bib-0020] Kanmani, P. , R. Satish kumar , N. Yuvaraj , K. A. Paari , V. Pattukumar , and V. Arul . 2011. “Production and Purification of a Novel Exopolysaccharide From Lactic Acid Bacterium *streptococcus phocae* PI80 and Its Functional Characteristics Activity In Vitro.” Bioresource Technology 102, no. 7: 4827–4833. 10.1016/j.biortech.2010.12.118. 21300540

[fsn34657-bib-0021] Kim, J. J. , M. S. Shajib , M. M. Manocha , and W. I. Khan . 2012. “Investigating Intestinal Inflammation in DSS‐Induced Model of IBD.” Journal of Visualized Experiments 60: e3678. 10.3791/3678. PMC3369627 22331082

[fsn34657-bib-0022] Kim, S. K. , R. B. Guevarra , Y. T. Kim , et al. 2019. “Role of Probiotics in Human Gut Microbiome‐Associated Diseases.” Journal of Microbiology and Biotechnology 29, no. 9: 1335–1340. 10.4014/jmb.1906.06064. 31434172

[fsn34657-bib-0023] Le Berre, C. , S. Honap , and L. Peyrin‐Biroulet . 2023. “Ulcerative Colitis.” Lancet 402, no. 10401: 571–584. 10.1016/s0140-6736(23)00966-2. 37573077

[fsn34657-bib-0024] Lee, H. , Y.‐T. Ahn , J.‐H. Lee , C.‐S. Huh , and D.‐H. Kim . 2009. “Evaluation of Anti‐Colitic Effect of Lactic Acid Bacteria in Mice by cDNA Microarray Analysis.” Inflammation 32, no. 6: 379–386. 10.1007/s10753-009-9146-y. 19711178

[fsn34657-bib-0025] Mulay, V. , D. Satav , A. Fernandez , P. Pisalwar , and S. Ahmed . 2022. “Development of Probiotics for *Helicobacter pylori* Infection Management.” In Alternatives to Antibiotics: Recent Trends and Future Prospects, edited by T. Saha , M. Deb Adhikari , and B. K. Tiwary , 499–523. Singapore: Springer Nature Singapore.

[fsn34657-bib-0026] Mustafa, A. , A. El‐Medany , H. H. Hagar , and G. El‐Medany . 2006. “ *Ginkgo biloba* Attenuates Mucosal Damage in a Rat Model of Ulcerative Colitis.” Pharmacological Research 53, no. 4: 324–330. 10.1016/j.phrs.2005.12.010. 16458529

[fsn34657-bib-0027] Ng, S. C. , H. Y. Shi , N. Hamidi , et al. 2017. “Worldwide Incidence and Prevalence of Inflammatory Bowel Disease in the 21st Century: A Systematic Review of Population‐Based Studies.” Lancet 390, no. 10114: 2769–2778. 10.1016/s0140-6736(17)32448-0. 29050646

[fsn34657-bib-0028] Niu, X. , Q. Li , N. Luan , et al. 2023. “ *Lactiplantibacillus plantarum* BW2013 Protects Mucosal Integrity and Modulates Gut Microbiota of Mice With Colitis.” Canadian Journal of Microbiology 69, no. 4: 158–169. 10.1139/cjm-2022-0092. 36669152

[fsn34657-bib-0029] Osman, N. , D. Adawi , S. Ahrné , B. Jeppsson , and G. Molin . 2008. “Probiotics and Blueberry Attenuate the Severity of Dextran Sulfate Sodium (DSS)‐Induced Colitis.” Digestive Diseases and Sciences 53, no. 9: 2464–2473. 10.1007/s10620-007-0174-x. 18274903

[fsn34657-bib-0030] Patel, M. , S. V. Subas , M. R. Ghani , et al. 2020. “Role of Substance P in the Pathophysiology of Inflammatory Bowel Disease and Its Correlation With the Degree of Inflammation.” Cureus Journal of Medical Science 12, no. 10: e11027. 10.7759/cureus.11027. PMC7671294 33214955

[fsn34657-bib-0031] Rodriguez‐Nogales, A. , F. Algieri , J. Garrido‐Mesa , et al. 2017. “Differential Intestinal Anti‐Inflammatory Effects of *Lactobacillus fermentum and Lactobacillus salivarius * in DSS Mouse Colitis: Impact on microRNAs Expression and Microbiota Composition.” Molecular Nutrition & Food Research 61, no. 11: 1700144. 10.1002/mnfr.201700144. 28752563

[fsn34657-bib-0032] Sim, I. , J. H. Koh , D. J. Kim , S. H. Gu , A. Park , and Y. H. Lim . 2015. “In Vitro Assessment of the Gastrointestinal Tolerance and Immunomodulatory Function of *Bacillus methylotrophicus* Isolated From a Traditional Korean Fermented Soybean Food.” Journal of Applied Microbiology 118, no. 3: 718–726. 10.1111/jam.12719. 25494714

[fsn34657-bib-0033] Sommer, J. , E. Engelowski , P. Baran , C. Garbers , D. M. Floss , and J. Scheller . 2014. “Interleukin‐6, but Not the Interleukin‐6 Receptor Plays a Role in Recovery From Dextran Sodium Sulfate‐Induced Colitis.” International Journal of Molecular Medicine 34, no. 3: 651–660. 10.3892/ijmm.2014.1825. 24993179 PMC4121342

[fsn34657-bib-0034] Song, S. , A. Jeong , J. Lim , B.‐K. Kim , D.‐J. Park , and S. Oh . 2023. “ *Lactiplantibacillus plantarum* L67 Probiotics vs Paraprobiotics for Reducing Pro‐Inflammatory Responses in Colitis Mice.” International Journal of Dairy Technology 76, no. 1: 168–177. 10.1111/1471-0307.12918.

[fsn34657-bib-0035] Sun, M. , Y. Liu , Y. Song , et al. 2020. “The Ameliorative Effect of *Lactobacillus plantarum* ‐12 on DSS‐Induced Murine Colitis.” Food & Function 11, no. 6: 5205–5222. 10.1039/d0fo00007h. 32458908

[fsn34657-bib-0036] Sun, M.‐C. , D.‐D. Li , Y.‐X. Chen , et al. 2023. “Insights Into the Mechanisms of Reuterin Against *Staphylococcus aureus* Based on Membrane Damage and Untargeted Metabolomics.” Food 12, no. 23: 4208. https://www.mdpi.com/2304‐8158/12/23/4208. 10.3390/foods12234208 PMC10706222 38231661

[fsn34657-bib-0037] Sun, M. C. , F. C. Zhang , X. Yin , et al. 2018. “ *Lactobacillus reuteri* F‐9‐35 Prevents DSS‐Induced Colitis by Inhibiting Proinflammatory Gene Expression and Restoring the Gut Microbiota in Mice.” Journal of Food Science 83, no. 10: 2645–2652. 10.1111/1750-3841.14326. 30216448

[fsn34657-bib-0038] Sun, X. , C. Y. Guo , F. Zhao , et al. 2019. “Vasoactive Intestinal Peptide Stabilizes Intestinal Immune Homeostasis Through Maintaining Interleukin‐10 Expression in Regulatory B Cells.” Theranostics 9, no. 10: 2800–2811. 10.7150/thno.34414. 31244924 PMC6568172

[fsn34657-bib-0039] Valatas, V. , M. Vakas , and G. Kolios . 2013. “The Value of Experimental Models of Colitis in Predicting Efficacy of Biological Therapies for Inflammatory Bowel Diseases.” American Journal of Physiology. Gastrointestinal and Liver Physiology 305, no. 11: G763–G785. 10.1152/ajpgi.00004.2013. 23989010

[fsn34657-bib-0040] Viennois, E. , F. Chen , H. Laroui , M. T. Baker , and D. Merlin . 2013. “Dextran Sodium Sulfate Inhibits the Activities of Both Polymerase and Reverse Transcriptase: Lithium Chloride Purification, a Rapid and Efficient Technique to Purify RNA.” BMC Research Notes 6: 360. 10.1186/1756-0500-6-360. 24010775 PMC3847706

[fsn34657-bib-0041] Wakuda, T. , K. Azuma , H. Saimoto , et al. 2013. “Protective Effects of Galacturonic Acid‐Rich Vinegar Brewed From Japanese Pear in a Dextran Sodium Sulfate‐Induced Acute Colitis Model.” Journal of Functional Foods 5, no. 1: 516–523. 10.1016/j.jff.2012.10.010.

[fsn34657-bib-0042] Wan, J. , J. Zhang , H. Yin , D. W. Chen , B. Yu , and J. He . 2020. “Ameliorative Effects of Alginate Oligosaccharide on Tumour Necrosis Factor‐α‐Induced Intestinal Epithelial Cell Injury.” International Immunopharmacology 89: 107084. 10.1016/j.intimp.2020.107084. 33242708

[fsn34657-bib-0043] Wang, D. , T. H. Zhang , H. W. Hao , H. X. Zhang , H. Q. Ye , and C. H. Zhao . 2022. “Probiotic Properties of a Spaceflight‐Induced Mutant *Lactobacillus Plantarum* SS18‐50 in Mice.” Endocrine, Metabolic & Immune Disorders Drug Targets 22, no. 5: 525–531. 10.2174/1871530321666210917163719. 34533451

[fsn34657-bib-0044] Wang, D. , T. H. Zhang , H. Q. Ye , H. W. Hao , H. X. Zhang , and C. H. Zhao . 2020. “In Vitro Probiotic Screening and Evaluation of Space‐Induced Mutant *Lactobacillus plantarum* .” Food Science & Nutrition 8, no. 11: 6031–6036. 10.1002/fsn3.1894. 33282255 PMC7684610

[fsn34657-bib-0045] Wang, Y. , Y. Gong , M. S. Farid , and C. Zhao . 2024. “Milk: A Natural Guardian for the Gut Barrier.” Journal of Agricultural and Food Chemistry 72, no. 15: 8285–8303. 10.1021/acs.jafc.3c06861. 38588092

[fsn34657-bib-0046] Youn, H. Y. , H. J. Kim , H. Kim , and K. H. Seo . 2024. “A Comparative Evaluation of the Kefir Yeast *Kluyveromyces marxianus* A4 and Sulfasalazine in Ulcerative Colitis: Anti‐Inflammatory Impact and Gut Microbiota Modulation.” Food & Function 15, no. 12: 6717–6730. 10.1039/d4fo00427b. 38833212

[fsn34657-bib-0047] Zhang, C. , Y. J. Hu , Y. Yuan , et al. 2023. “Liposome‐Embedded SOD Attenuated DSS‐Induced Ulcerative Colitis in Mice by Ameliorating Oxidative Stress and Intestinal Barrier Dysfunction.” Food & Function 14, no. 9: 4392–4405. 10.1039/d2fo03312g. 37092895

[fsn34657-bib-0048] Zhang, Z. , P. Shen , J. Liu , et al. 2017. “In Vivo Study of the Efficacy of the Essential Oil of *Zanthoxylum bungeanum* Pericarp in Dextran Sulfate Sodium‐Induced Murine Experimental Colitis.” Journal of Agricultural and Food Chemistry 65, no. 16: 3312–3320. 10.1021/acs.jafc.7b01323. 28368613

[fsn34657-bib-0049] Zheng, B. , J. Van Bergenhenegouwen , H. J. G. Van de Kant , et al. 2016. “Specific Probiotic Dietary Supplementation Leads to Different Effects During Remission and Relapse in Murine Chronic Colitis.” Beneficial Microbes 7, no. 2: 205–213. 10.3920/bm2015.0037. 26645352

[fsn34657-bib-0050] Zhu, H. , and Y. R. Li . 2012. “Oxidative Stress and Redox Signaling Mechanisms of Inflammatory Bowel Disease: Updated Experimental and Clinical Evidence.” Experimental Biology and Medicine 237, no. 5: 474–480. 10.1258/ebm.2011.011358. 22442342

[fsn34657-bib-0051] Zizzo, M. G. , G. Caldara , A. Bellanca , et al. 2020. “AphaMax(R), an Aphanizomenon Flos‐Aquae Aqueous Extract, Exerts Intestinal Protective Effects in Experimental Colitis in Rats.” Nutrients 12, no. 12: 3635. 10.3390/nu12123635. 33256017 PMC7760929

